# Determining cost-saving risk thresholds for statin use

**DOI:** 10.1371/journal.pone.0318454

**Published:** 2025-03-13

**Authors:** Afschin Gandjour

**Affiliations:** Frankfurt School of Finance & Management, Frankfurt, Germany; University of Messina, ITALY

## Abstract

**Background:**

The German government has recently drafted a bill proposing a reduction in the prescription threshold for statin use. This study aims to determine the cost-saving risk threshold for statin use in Germany to inform this proposed change.

**Methods:**

An economic evaluation utilizing a decision-analytic model was performed, using secondary data to compare statin use versus no statin use from the perspective of German sickness fund insurees. The analysis focused on cost savings from avoided cardiovascular (CV) events, translating these avoided events into net savings after accounting for treatment costs and potential side effects. The study considered the German adult population insured by sickness funds and used a lifetime horizon for the analysis.

**Results:**

The maximum number needed to treat (NNT) to achieve cost savings over 10 years was found to be 39, leading to a minimum CV risk threshold for savings of 10.2%. It was estimated that approximately 19% of the adult population in Germany has a 10-year CV risk of ≥ 10.2%, potentially avoiding between 271,739 and 581,363 CV events over 10 years, with net population savings of approximately €15 billion.

**Conclusions:**

A threshold for statin prescription in Germany set at a 10.2% 10-year CV risk could significantly increase the number of patients benefiting from statin therapy, reducing CV events and generating substantial cost savings. These findings suggest that adjustments to prescription guidelines could improve cardiovascular outcomes and economic efficiency within the German healthcare system.

## Introduction

According to their label, statins should be prescribed alongside a diet and other non-pharmacological measures when these alone do not sufficiently lower cholesterol levels. In Germany, approximately 4 to 6 million people use statins to reduce their cholesterol levels and decrease the risk of cardiovascular (CV) diseases [1]. Statin adherence in Germany has been reported at 84% in two recent studies: Salam et al. [2] collected self-reported data from 74 patients with coronary heart disease, while Koenig et al. [3] analyzed prescription data from sickness funds covering 865,732 statin users. Despite this, a significant number of patients who could benefit from statin therapy remain untreated [4,5]. Internationally, Germany ranked only 25th out of 91 countries in statin utilization among individuals over 40 years old as of mid-2020 [6].

Overuse of statins may also be a concern, particularly in individuals with a low baseline risk of cardiovascular disease, where the absolute benefit of risk reduction is minimal. For these individuals, the potential for side effects may outweigh the small potential benefit. Overuse can also occur when prescribing practices do not align with clinical guidelines, which often recommend statins primarily for individuals with specific risk profiles. Statin intolerance is not negligible and was observed in 12.5% of participants in a German study, though these results carry some uncertainty due to the uncontrolled nature of the study [7].

In 2021, the number of hospital admissions with ischemic stroke in Germany was 216,923 [8]. Approximately 80% of strokes are ischemic [9]. Additionally, around 280,000 people in Germany suffer a heart attack each year [10]. A recent commentary by Baldus and Lauterbach [11] emphasizes that Germany can learn from other developed countries in tackling cardiovascular morbidity and mortality. Primary prevention of cardiovascular disease will remain crucial, requiring systematic identification and control of cardiovascular risk factors.

Statin trials suggest that reducing low-density lipoprotein (LDL) cholesterol with statins lowers the risk of major vascular events, largely irrespective of age, sex, baseline LDL cholesterol, or previous vascular disease, and reduces vascular and all-cause mortality [12]. Consequently, the number needed to treat is lower in higher-risk groups, making statins more cost-effective in these groups due to the greater number of prevented events per 100 treated patients [13].

In 2020, the German Federal Joint Committee (G-BA) [14] had specified that, outside of secondary prevention, lipid-lowering drugs could only be prescribed for patients with a high cardiovascular risk, defined as a 20% or greater event rate over 10 years according to validated risk calculators. The justification for this restriction was that, except in cases of secondary prevention, lifestyle changes (e.g., weight reduction and dietary measures) were considered the first-line therapy for hyperlipidemia [14]. However, the rationale for the specific 20% threshold was not made public.

On August 28, 2024, the German government drafted the Healthy Heart Act [15]. To prevent severe cardiovascular events such as heart attacks and strokes, the prescription of statins will be strengthened based on current scientific knowledge and medical guidelines for specific risk profiles. The specifics, particularly regarding risk thresholds, will be established by G-BA. This change will allow contracted physicians to prescribe lipid-lowering agents to patients earlier than before, in alignment with their individual cardiovascular risk. However, the law is not yet finalized and still requires further legislative approval.

On December 19, 2024, the G-BA revised its decision rule, lowering the prescription threshold to a 10% event rate over 10 years [16]. This decision was primarily supported by a guideline from the National Institute for Health and Care Excellence [17]. The Institute for Quality and Efficiency in Health Care (IQWiG) was not involved in the decision-making process.

Given the recent legislative proposal and the change in the prescription threshold established by the G-BA, this study aims to identify a prescription threshold based on the economic benefits of statins. While cost considerations in reimbursement decisions are mandated by law (Social Code Book V § 12), they were not explicitly addressed by the G-BA. Therefore, providing evidence on the economic benefits of statins is crucial to informing this policy. Specifically, the study focuses on determining the risk threshold for statin prescriptions in Germany that is predicted to yield cost savings for sickness funds. These funds insure approximately 87% of the German population [18].

While several cost-effectiveness analyses on statin use for preventing cardiovascular disease in Germany have been conducted, mostly before statins became generic [19,20], there is a lack of information on the current cost savings potential of statins.

## Methods

### Conceptual approach

The analysis was conducted from the perspective of German sickness fund insurees. This perspective is relevant for decisions by the G-BA, which assesses the annual treatment costs of new innovative drugs. Unlike the sickness funds’ perspective, this specific viewpoint includes copayments made by the insurees but excludes direct non-medical costs and indirect costs.

The study considered the German adult population insured by sickness funds, comparing statin use to no statin use.

### Methodological approach

An economic evaluation using a decision-analytic model and secondary data was conducted. The analysis assessed the health benefits of statin use in terms of avoided CV events and translated these benefits into cost savings, effectively representing a cost analysis.

The measure of benefits was the net savings from avoided CV events, accounting for healthcare costs during additional life years. The harms of statin use were also considered in terms of costs. The analysis was conducted over a lifetime horizon based on the underlying data sources (see Cost Analysis).

The study determined the threshold for the 10-year risk of CV events based on the point of cost neutrality, at which interventions costs equal cost offsets.

The maximum number needed to treat (NNT) to achieve savings over 10 years was calculated as follows:


$$NNT_{\text{max}}=\frac{C_{\text{CV}}}{C_{\text{T}}},$$ (1)

where $C_{\text{CV}}$ is the weighted average of the lifetime costs of strokes and myocardial infarctions (MIs), and $C_{\text{T}}$ is the treatment cost over 10 years. The formula for $C_{\text{CV}}$ is:


$$C_{\text{CV}}=\frac{\left(C_{\text{stroke}}\times ARR_{\text{stroke}}\right)+\left(C_{\text{MI}}\times ARR_{\text{MI}}\right)}{ARR_{\text{stroke}}+ARR_{\text{MI}}},$$ (2)

where $C_{\text{stroke}}$ is the lifetime cost of stroke, $C_{\text{MI}}$ is the lifetime cost of MI, $ARR_{\text{stroke}}$ is the absolute risk reduction for stroke, and $ARR_{\text{stroke}}$ is the absolute risk reduction for MI.

Next, the minimum absolute risk reduction ($ARR_{\text{min}}$) required to achieve the maximum NNT for cost savings was calculated based on the method described by Cook and Sackett [21]. This was followed by the calculation of the minimum baseline 10-year risk for CV events ($P_{\text{min}}$) using the relative risk reduction (RRR) of statins [21]:


$$ARR_{\text{min}}=\frac{1}{NNT_{\text{max}}},$$ (3)

$$P_{\text{min}}=\frac{ARR_{\text{min}}}{RRR}.$$ (4)

From the risk distribution in the German population, the fraction with a risk above $P_{\text{min}}$ was determined. While $P_{\text{min}}$ defines the minimum threshold for cost savings, the average risk in the population at or above $P_{\text{min}}$ is actually higher. Considering the average risk ($P_{\text{avg}}$) is necessary to calculate the number of avoided CV events in the population at or above $P_{\text{min}}$. This was determined by subtracting expected CV events with the relative risk reduction from the initial expected CV events based on $P_{\text{avg}}$:


$$E_{0}=N\times P_{\text{avg}},$$ (5)

$$E_{\text{t}}=N\times P_{\text{avg}}\times \left(1-RRR\right),$$ (6)

$$�E=E_{0}-E_{\text{t}},$$ (7)

where $E_{\text{t}}$ and $E_{0}$ are the number of expected CV events with and without treatment, respectively, and *N* is the total number of individuals in the population segment.

### Epidemiological data

Based on the meta-analysis by Byrne et al. [22], a combined RRR for both stroke and myocardial infarction was calculated using a weighted average approach. This involves weighting each RRR by its respective ARR and summing these weighted values. Since ARR increases with baseline risk, higher-risk patients, such as those with elevated LDL-C or previous cardiovascular events, experience a greater absolute benefit from treatment, despite the RRR remaining consistent across different risk groups [23,24]. The combined RRR for stroke and MI, considering their respective ARRs, is approximately 25.57%. This result is corroborated by a recent systematic review of RCTs showing a median reduction of major CV events by 26% [25]. Most clinical trials report results based on an intention-to-treat (ITT) analysis, which includes all participants as originally allocated after randomization, regardless of their adherence to the treatment regimen. Therefore, the reported RRR typically includes some level of non-adherence.

Using data from the German Health Examination Survey for Adults (DEGS1), we estimated the average 10-year CV risk for individuals in Germany. The DEGS1 survey categorizes individuals into low (<1%), moderate (1-<5%), and high-risk (≥5%) groups based on the SCORE system for CV disease mortality risk. The prevalence of low, moderate, and high risk was 42.8%, 38.5%, and 18.8% in men and 73.7%, 18.1%, and 8.2% in women, respectively [26]. The SCORE model and related literature often suggest that the incidence of non-fatal CV disease events is approximately 3-5 times higher than that of fatal events.

### Cost analysis

For the cost of statins, the analysis considered the pharmacy retail price after legally mandated discounts from the reference price. Atorvastatin was selected as it is frequently used for the prevention of CV diseases [7], with 40 mg being the most commonly recommended dose for adults, especially for the prevention of CV events. In a sensitivity analysis, the cheapest statin dose was assumed.

The annual total costs for the treatment and monitoring of a patient taking statins for the prevention of cardiovascular diseases, excluding medication costs, are based on the public physician reimbursement scheme in Germany [27] and range from €31 to €62. These costs include regular doctor visits for general and extended examinations (EBM codes 03220 and 03230), costing approximately €15 per visit, occurring 1-2 times per year. Laboratory tests such as lipid profiles (EBM codes 32060, 32061, 32062, 32063) cost €0.15 per test, performed 1-2 times per year, and liver function tests (EBM code 32070) cost €0.25 per test, conducted as needed. Additional specific tests like creatine kinase determination (EBM code 32074) can cost €0.25 per test and are required as needed [28, p. 162].

It was estimated that 12% of statin users in Germany require additional clinical visits annually due to side effects (based on [7]), with the cost per patient ranging from €19 to €60 based on the type and severity of the side effects. These additional visits are due to issues such as severe muscle pain, new-onset diabetes, significant liver enzyme elevation, and severe gastrointestinal problems.

The data on savings were derived from the lifetime costs of ischemic stroke survivors in Germany, including nursing care, as reported by Kolominsky-Rabas et al. [29]. That study utilized medical records and interviews. It described survival within the first year after an ischemic stroke using nonparameterized Kaplan-Meier curves. Survival from years 2 to 10 was modeled using a Weibull parameterization.

In the present modeling study, costs were inflated to 2024 euros to account for inflation. This study also considered the increase in health expenditures for treating strokes due to technological advancements, reflecting the decrease in case fatality rates (CFR) of strokes in Germany over time [30]. Improvements in stroke management and treatment, such as increased stroke units and uptake of acute therapies like thrombolytic therapy, contributed to this decrease. The present study estimated the cost impact of higher long-term care costs for stroke survivors due to reduced CFR, including rehabilitation costs, outpatient services, and long-term care facilities. Assuming these advancements have added approximately 35% to the costs over 20 years, the estimated inflated expenditure for stroke care in 2024, considering inflation and technological advancements, is approximately €90,245.

This estimate was confirmed by a recently published long-term cost analysis using the InGef (Institute of Applied Health Research) research database with sickness fund claims data for the period from 2014 to 2018 [31]. The study reported costs in the first year after an ischemic stroke of €22,404, in addition to a matched control group. After the 6th quarter, insured individuals with an incident ischemic stroke incur approximately €700 per quarter in additional medical treatment costs compared to the control group. Incorporating the control group’s cost of approximately €5,000 per year, the discounted remaining life expectancy of 5.9 years reported by Kolominsky-Rabas et al. [29], and inflation from 2018 to 2024 yields costs of survivors of about €84,000. It is important to note that discounting future costs in addition to using the discounted remaining life expectancy results in double discounting and is therefore inappropriate. As the analysis has not been peer-reviewed and is limited to a 4-year observational period, its estimate was considered more uncertain and was thus considered the lower limit in a sensitivity analysis.

It would have been possible to consider only the costs of ischemic strokes on top of those of the control group as a saving. However, this approach would have required considering general health expenditures (those of the control group) in added years of survival, necessitating additional investigation into survival gains from stroke prevention.

The lifetime cost of MI was predicted to be lower than that of ischemic stroke due to several factors: lower initial costs related to acute care, hospitalization, and follow-up treatments, and lower long-term costs such as medication, lifestyle management, and monitoring. This is because MI typically involves shorter rehabilitation periods and lower rates of long-term disability [32]. The study conservatively estimated that the lifetime cost of MI might range between 60% to 80% of the lifetime cost of ischemic stroke. This range was applied in a sensitivity analysis.

The RRR for all-cause mortality is about one-third of that for CV events [25]. Assuming an approximately constant hazard rate, as in an exponential survival model, each euro saved by avoiding cardiovascular events is offset by approximately one-third of a euro in life extension costs. This proportional relationship arises because, in an exponential survival model, a one-third reduction in all-cause mortality translates to a one-third extension in remaining life expectancy [33]. Over intermediate time frames, such as 10 years, the assumption of a relatively stable hazard rate in this population appears plausible, particularly in individuals at moderate-to-high risk of cardiovascular events. Life extension costs include both CV-related and non-CV-related expenditures. Since the effect of statins on all-cause mortality, not just CV mortality, is considered, non-CV mortality is also taken into account.

This may present an overestimation of savings because, in patients with CV events (MIs and strokes), not all healthcare expenditures are driven by CV events—approximately 80% of them are [34]. Conversely, it may present an underestimation of savings because, in populations at higher risk (such as older adults or those with existing CV conditions), the hazard rate generally increases over time. Additional uncertainties arise from the dependence of the RRR for all-cause mortality on baseline LDL-C levels [24]. For LDL-C levels <100 mg/dL, the relative risk for all-cause mortality is 1.0, whereas for LDL-C levels >100 mg/dL, it is 0.85 (95% confidence interval: 0.80–0.91) when combining the three LDL-C categories (100–129, 130–159, and >160 mg/dL) using a meta-analytic approach. This indicates that for patients with LDL-C levels >100 mg/dL, each euro saved from avoided CV events is offset by approximately three-fifths of a euro in life extension costs. Nevertheless, under the exponential survival model, the results are equivalent whether the average life extension is computed using the average risk ratio or the weighted average of life extensions across subgroups with different risk ratios.

The finding that only a portion of cost savings from statins would be offset by life-prolonging costs is consistent with the results of a modeling study using Dutch data. This study suggested that preventing “diseases of the circulatory system” would increase life expectancy while reducing healthcare spending, indicating that savings from avoiding these diseases would not be entirely offset by life extension costs [35]. However, given the uncertainties mentioned above, a sensitivity analysis was conducted in which the fraction of offsets was varied between 20% and 50%. For a discussion of life extension costs associated with statin therapy from various health economic perspectives in Germany, see Wendland et al. [36].

Discounting was not directly applied as the estimates of the lifetime costs of CV events already incorporated discounting [29].

### Sensitivity analysis

In deterministic one-way analyses, uncertainty in the minimum CV risk was assessed by varying individual input parameters susceptible to variation, one at a time, using the boundaries described in Table 1. Furthermore, scenario analysis was provided for the number of CV events avoided and population/individual costs saved (Tables 2 and 3).

**Table 1:** Base-case values and ranges. All costs are in euros.

Variable	Mean (range)	Reference
Cost data		
Cost of a statin per day	0.19 (0.12 − 0.19)	[37,38]
Ancillary costs (doctor visits, lab tests)	47 (31 − 62)	[27,28]
Costs of side effects per year	40 (19 – 60)	Assumption
Lifetime cost for stroke care	90,245 (72,780 − 113,193)	[29]
Lifetime cost for MI care	63,172 (54,147 − 72,196)	Assumption
Fraction of cost savings offset by life-prolonging costs	0.33 (0.20 − 0.50)	[33]
Epidemiological data		
German adult population with social health insurance	60,500,000	[18,39]
Reduction in the number of non-fatal MIs compared to non-fatal strokes	3.25 (1.92 − 4.58)	[22]
Relative risk reduction of CV events	0.25 (0.20 – 0.30)	[22]
Prevalence of 10-year CV disease risk ≥ 10.2%	0.19 (0.18 – 0.21)	[26]

MI: myocardial infarction; CV: cardiovascular.

**Table 2:** Avoided cardiovascular events over 10 years in sickness fund insurees.

Scenario	Base case	Low estimate	High estimate
Population size (10-year CVD risk ≥ 10.2%)	11,591,589	10,708,305	12,462,773
Initial expected CV events (14%)	1,626,300	1,335,326	1,915,528
Expected CV events (25% risk reduction)	1,212,072	1,063,587	1,334,165
Avoided CV events	414,228	271,739	581,363

CV: cardiovascular.

**Table 3:** Population and individual savings from statin use in the population above the risk threshold over a 10-year period. All costs are in euros.

Scenario	Base case	Low estimate	High estimate
Intervention cost	13,935,814,290	12,873,899,422	14,983,182,379
Savings	28,806,145,675	18,897,185,893	40,428,980,063
Net population saving	14,870,331,385	6,023,286,470	25,445,797,685
Net individual saving	1,283	562	2,042

## Results

The maximum NNT reaching the cost-saving threshold was 39, reflecting the ratio of the weighted-average lifetime cost of CV events to the 10-year cost of statin therapy (€46,361/€1202). The resulting minimum risk threshold for savings ($P_{\text{min}}$ in Equation 4) was 10.2%, calculated as the ratio of the $ARR_{\text{min}}$ to the RRR (2.6%/25%).

**Fig 1:**
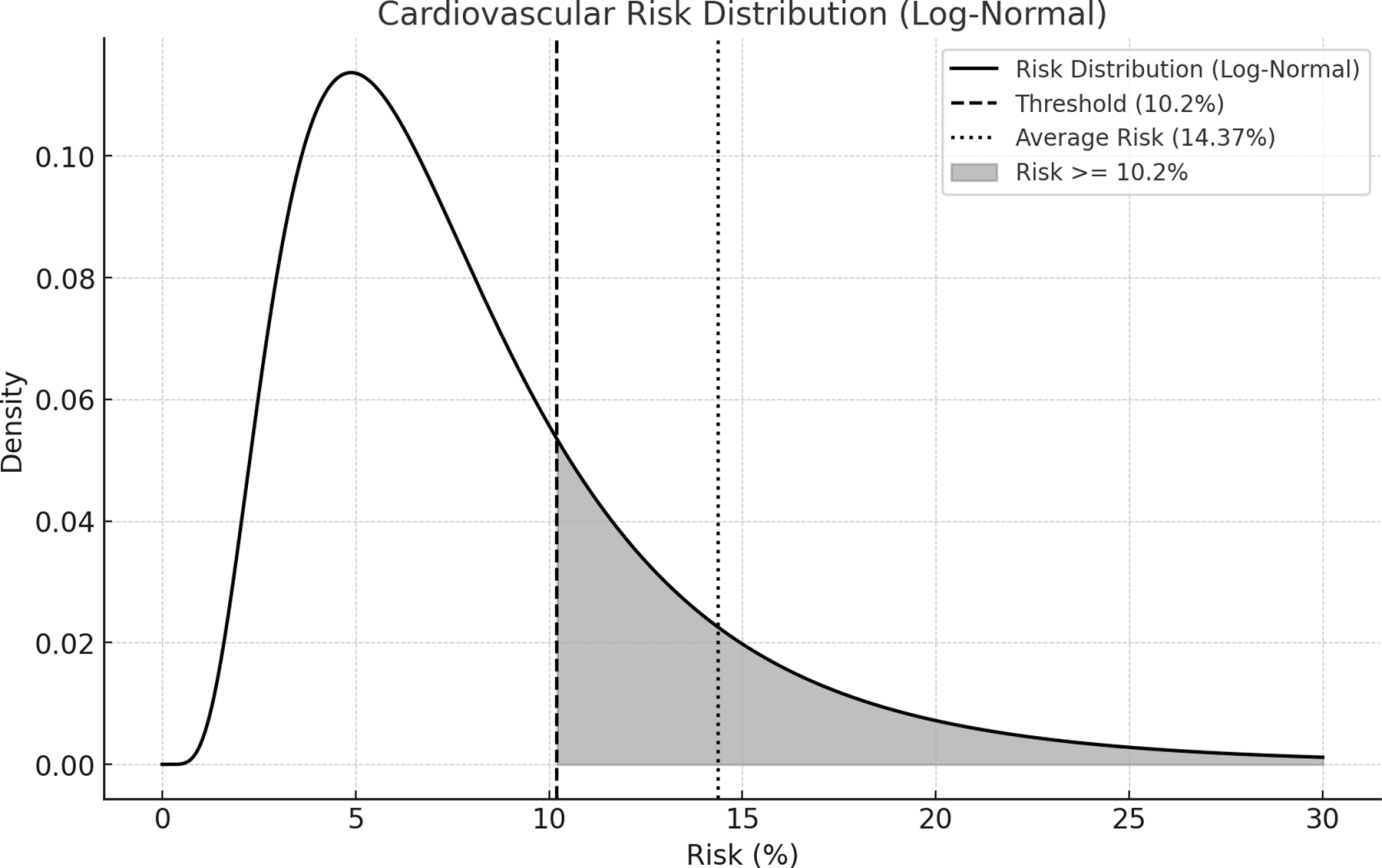
Estimated distribution of 10-year cardiovascular risk in the German population. Dashed lines indicate minimum cost-saving threshold and average risk with a risk above the threshold.

Using data from the German Health Examination Survey for Adults (DEGS1), we estimated the average 10-year CV risk for individuals in Germany with a minimum risk of 10.2%. Given that more than 50% of the population falls into the low-risk category [26], we focused on the moderate and high-risk groups. Using a log-normal distribution to model the right-skewed nature of CV risk, we calculated an average risk of approximately 14.37% for individuals above the 10.2% threshold (see Fig 1). This aligns well with the DEGS1 data, suggesting that our model accurately reflects the distribution of CV risk in the German population.

Based on the risk distribution curve, we assumed that around 19% of the adult population in Germany might have a 10-year CVD risk of ≥10.2% (Fig 1). Assuming that the average risk above that threshold ranges between 12% and 15%, the number of avoided CV events over 10 years is estimated to be between 271,739 and 581,363 among adults insured by sickness funds, with a base-case estimate of 414,228, or 41,423 per year (Table 2). The resulting net population savings for sickness funds are approximately €15 billion over 10 years, or €1.5 billion annually (Table 3).

### Sensitivity Analysis

**Fig 2:**
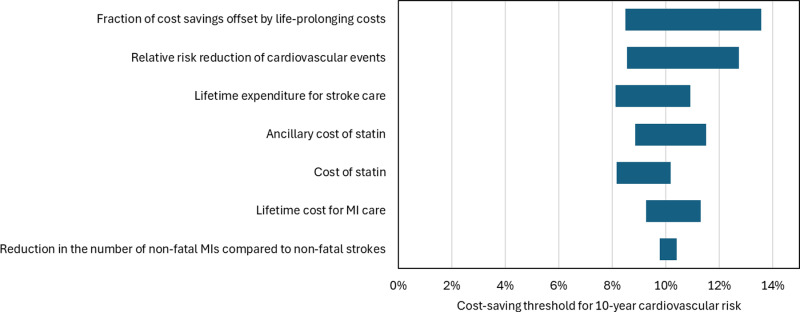
One-way sensitivity analysis of the cost-saving threshold for 10-year cardiovascular risk. Variables are arranged in order of their impact on the 10-year risk threshold. MI: myocardial infarction.

The variable with the greatest impact on the cost-saving threshold for 10-year CV risk was the fraction of cost savings offset by life-prolonging costs, followed by the RRR of CV events (Fig 2). The one-way sensitivity analysis indicated that the cost-saving threshold for 10-year CV risk consistently remains at or below 14% (Fig 2).

## Discussion

The results of this analysis support the revised official threshold for statin prescriptions in Germany, established by the G-BA at a 10% 10-year risk in December 2024 [16]. The risk threshold for statin use determined in this study also aligns with the recommendations of the European Society of Cardiology (ESC) for individuals aged 50 to 69 years, which is a 10% CV disease risk [40]. Similar to the ESC recommendations, this risk threshold does not differentiate between primary and secondary prevention. Additionally, a 10% 10-year cardiovascular risk can be reached through different combinations of age, sex, and other cardiovascular risk factors. For example, younger individuals with multiple high-risk factors may reach the same 10% risk as older individuals with fewer risk factors. Decisions to initiate statin therapy should prioritize optimizing patient health outcomes over cost considerations alone, ensuring that financial incentives do not compromise clinical judgment. The justification for a 10% threshold, therefore, lies not only in its potential to generate savings but also in its capacity to prevent more cardiovascular events.

A significant portion of the population could benefit from statin therapy under the new threshold. A population of 12 million sickness fund insurees who would have an indication for statins based on the cost-saving threshold is more than twice as large as the current number of statin users. The uptake rate represents the upper bound of the overuse rate, even in the presence of off-label use [41]. This indicates that the degree of statin underuse must be larger than the degree of statin overuse in sickness fund insurees.

The rate of underuse is relevant when interpreting the number of avoided CV events and population savings. Although underuse extends above 50% for the newly defined 10-year risk threshold, a portion of CV events is avoided already today by statin use. Hence, the total number of CV events avoided and costs saved reflect the maximum benefit rather than an additional benefit over current practice. Assuming that only 40% of patients are already taking statins above the threshold (based on a total prescription number of about 5 million), we obtain approximately 28,000 CV events avoided per year and savings of €1 billion per year (€1.5 billion minus €500 million). Based on the incidence of MIs and ischemic strokes in Germany, additional statin prescriptions for individuals above the risk threshold have the potential to reduce this clinical burden by approximately 2%.

The risk threshold for older adults might need adjustment due to different risk-benefit profiles and potential polypharmacy issues. Ensuring that older adults are not overprescribed statins due to competing risks is critical. Although the lifetime costs used in this study were based on a median age of approximately 75 years [29], it is prudent to use the threshold cautiously for patients above this age. On the one hand, despite a shorter remaining life expectancy of stroke and MI survivors above the age of 75, most expenditures for stroke and MI occur in the first year after the incident, which is relatively unaffected by the age of the incident [31]. On the other hand, based on a registry analysis of stroke patients with a mean age of 76 years from Sweden, mortality after ischemic stroke increases by 6% with each additional year [42]. Assuming a proportional reduction in expenditure, this would lead to a proportional increase in the cost-saving risk threshold by 0.6% per year. Therefore, the sensitivity of the threshold to the age of stroke or MI incidence is limited. For instance, to increase the 10-year risk threshold from 10.2% to 16.2%, the age of incidence would need to increase from 75 to 85 years.

While it is possible to calculate gender-specific risk thresholds based on gender-specific healthcare savings, this approach may be ethically controversial.

The savings per individual from statin use, as calculated in this analysis, can serve as a benchmark for the upper limit of a reward to users of statins. The annual reward could be as high as €128, which is larger than the cost of the statin itself. Ensuring that patients adhere to their medication to qualify for the reward involves a multifaceted approach combining technology and behavioral strategies. Smart pill bottles provide dose reminders and log each time the bottle is opened. To confirm ingestion, additional methods such as video verification, cholesterol level checks, ingestible sensors, and wearable devices monitoring physiological changes post-ingestion can be employed.

Alternatively, the analysis allows determining the maximum annual costs of statins at which they can be made available without copayments while remaining cost-neutral for sickness fund insurees. This is calculated as the annual cost based on the daily cost of 19 cents plus the annual savings of statins, totaling approximately €197. Currently, a medication can be exempt from copayments if its price is at least 30% below the reference price, which is the maximum amount statutory health insurance covers for certain medication groups.

A cost-effectiveness analysis by Lazar et al. [43] using U.S. data identified several scenarios where statin use is cost-saving. The scenario closest to this study involves a 10%-20% risk of coronary heart disease (CHD) over 10 years, two risk factors, and an LDL cholesterol level of > 100 mg/dL. Notably, the 10-year risk in this scenario refers specifically to CHD, not CV disease as in this paper. This increases the cost-saving treatment threshold because CHD is less expensive than strokes, but additional risk factors included in the scenario increase CV risk, thus lowering the cost-saving treatment threshold.

Previous research in Germany includes a cost-effectiveness analysis that examined the cost-effectiveness of statin therapy in primary prevention for different baseline risks [20]. The study concluded that statin therapy is cost-effective even at a 5-year risk of 7.5% from the perspective of statutory health insurance. The least favorable cost-effectiveness ratio was found at a 5-year risk of 7.5%, with €26,000 per life year gained for 70-year-old women. From the perspective of social health insurance, pension expenditures significantly increase the cost-effectiveness ratio. However, this study was conducted before generic statins became available in 2003 [44], potentially underestimating the cost-effectiveness of statins due to the drop in prices.

Additionally, a recent cost-effectiveness analysis compared additive lipid-lowering therapies to statin monotherapy for primary and secondary CV prevention in Germany [45].

The main limitation of this study lies in its modeling assumptions, although extensive sensitivity analysis was conducted to test the range of possible values. The study did not use a Markov model, which is typically used for cost-effectiveness modeling and often provides greater precision of estimates. However, Markov models carry assumptions such as a finite set of health states and memoryless transitions occurring at discrete intervals. These assumptions were avoided by using longitudinal cost data from the literature that accounted for the true curvilinear shape of the survival curve in each year post-stroke [29]. Moreover, the approach taken in this study allows for directly comparing savings, life extension costs, and intervention costs, thus enabling a direct inference of the NNT, which is key to defining the risk threshold indicating savings potential. This also leads to more transparent calculations, which is crucial for convincing policymakers and changing health policy.

Costs of implementation, such as those for public health campaigns, were not considered in the analysis. These may also lead to spillover effects on lower-risk subgroups without cost savings.

The generalizability of this study’s findings to other healthcare systems depends on similarities in population demographics, healthcare infrastructure, and economic conditions. While countries with comparable healthcare systems, cost structures, and guidelines may find the results more applicable, significant differences in medication costs, healthcare policies, insurance models, and epidemiology of CV diseases can affect the relevance and applicability of the study’s conclusions. Therefore, each country should consider local data and context-specific factors to determine the cost-effectiveness and appropriate use of statins for CV prevention within their healthcare system.

Future research should investigate factors influencing the 10-year risk threshold, as highlighted in the sensitivity analysis. Additionally, since combination therapy with statins and other cholesterol-lowering agents, such as PCSK9 inhibitors and ezetimibe, is warranted for patients who do not achieve optimal LDL cholesterol reduction with statin monotherapy [40], further analysis of the risk threshold for combination therapies is recommended.

## Conclusions

This study demonstrates that a cost-saving threshold for statin prescription in Germany is achievable at a 10.2% 10-year CV risk, significantly lower than the past threshold of 20% specified by the G-BA and in agreement with the recently revised threshold of 10%. By reducing the threshold, statin therapy could be extended to a larger portion of the population, thereby preventing more cardiovascular events and generating substantial economic savings for sickness funds. Specifically, the findings highlight that over 41,000 cardiovascular events could be avoided annually among adults insured by sickness funds, translating to approximately €1.5 billion in cost savings per year.

## Supporting information

S1 Checklist PLOS ONE human subjects research checklist.
(DOCX)

## References

[1] Laufs U, Scharnagl H, Halle M, Windler E, Endres M, März W. Treatment options for statin-associated muscle symptoms. Dtsch Arztebl Int. 2015;112(44):748–55. doi: 10.3238/arztebl.2015.0748 26575138 PMC4650909

[2] Salam B, Schrimpf A, Münster S, Bleckwenn M. Statin adherence in patients enrolled in the disease management program for coronary artery disease - comparison between patients’ and general practitioners’ self-reports and patient records. Res Health Serv Reg. 2023;2(1):13. doi: 10.1007/s43999-023-00029-3 39177923 PMC11281732

[3] Koenig W, Lorenz ES, Beier L, Gouni-Berthold I. Retrospective real-world analysis of adherence and persistence to lipid-lowering therapy in Germany. Clin Res Cardiol. 2024;113(6):812–21. doi: 10.1007/s00392-023-02257-6 37603070 PMC11108924

[4] Scheidt-Nave C, Du Y, Knopf H, Schienkiewitz A, Ziese T, Nowossadeck E, et al. Prevalence of dyslipidemia among adults in Germany: results of the German Health Interview and Examination Survey for Adults (DEGS 1). Bundesgesundheitsblatt Gesundheitsforschung Gesundheitsschutz. 2013;56(5–6):661–7. doi: 10.1007/s00103-013-1670-0 23703484

[5] Knopf HC, Busch MA, Du Y, Truthmann J, Schienkiewitz A, Scheidt-Nave C. Changes in the prevalence of statin use in Germany - findings from national health interview and examination surveys 1997-1999 and 2008-2011. Z Evid Fortbild Qual Gesundhwes. 2017;122:22–31. doi: 10.1016/j.zefq.2017.04.001 28511896

[6] Guadamuz J, Shooshtari A, Qato D. Global, regional and national trends in statin utilisation in high-income and low/middle-income countries, 2015-2020. BMJ Open. 2022;12(9):e061350. doi: 10.1136/bmjopen-2021-061350 PMC9462115 36691204

[7] Parhofer KG, Anastassopoulou A, Calver H, Becker C, Rathore AS, Dave R, et al. Estimating Prevalence and Characteristics of Statin Intolerance among High and Very High Cardiovascular Risk Patients in Germany (2017 to 2020). J Clin Med. 2023;12(2):705. doi: 10.3390/jcm12020705 36675634 PMC9864390

[8] Ungerer MN, Bartig D, Richter D, Krogias C, Hacke W, Gumbinger C. The evolution of acute stroke care in Germany from 2019 to 2021: analysis of nation-wide administrative datasets. Neurol Res Pract. 2024;6(1):4. doi: 10.1186/s42466-023-00297-x 38200611 PMC10782681

[9] Seite „Ischämischer Schlaganfall“. In: Wikipedia – Die freie Enzyklopädie. Bearbeitungsstand: 5. Juni 2024, 15:16 UTC. URL: https://de.wikipedia.org/w/index.php?title=Isch%C3%A4mischer_Schlaganfall&oldid=245659206 (Abgerufen: 25. Juni 2024, 14:17 UTC).

[10] Seite „Herzinfarkt“. In: Wikipedia – Die freie Enzyklopädie. Bearbeitungsstand: 6. Mai 2024, 13:48 UTC. URL: https://de.wikipedia.org/w/index.php?title=Herzinfarkt&oldid=244725906 (Abgerufen: 21. Juni 2024, 14:40 UTC).

[11] Baldus S, Lauterbach K. Prevention-centered health care in Germany - a nation in need to turn the tide. Eur J Epidemiol. 2023;38(8):835–7. doi: 10.1007/s10654-023-01030-3 37524897 PMC10421807

[12] Cholesterol Treatment Trialists’ (CTT) Collaborators, Mihaylova B, Emberson J, Blackwell L, Keech A, Simes J, et al. The effects of lowering LDL cholesterol with statin therapy in people at low risk of vascular disease: meta-analysis of individual data from 27 randomised trials. Lancet. 2012;380(9841):581–90. doi: 10.1016/S0140-6736(12)61067-8 22607822 PMC3437972

[13] Heller DJ, Coxson PG, Penko J, Pletcher MJ, Goldman L, Odden MC, et al. Evaluating the impact and cost-effectiveness of statin use guidelines for primary prevention of coronary heart disease and stroke. Circulation. 2017;136(12):1087–98. doi: 10.1161/CIRCULATIONAHA.117.027067 28687710 PMC5605438

[14] Gemeinsamer Bundesausschuss. Tragende Gründe zum Beschluss des Gemeinsamen Bundesausschusses über eine Änderung der Arzneimittel-Richtlinie (AM-RL): Anlage III (Verordnungseinschränkungen und -ausschlüsse) – Nummer 35 Lipidsenker. Vom 20. November 2020. https://www.g-ba.de/downloads/40-268-7102/2020-11-20_AM-RL-III_Nr35-Lipidsenker_TrG.pdf

[15] Bundesregierung. Entwurf eines Gesetzes zur Stärkung der Herzgesundheit (Gesundes Herz-Gesetz – GHG). 28.08.2024. https://www.bundesgesundheitsministerium.de/fileadmin/Dateien/3_Downloads/Gesetze_und_Verordnungen/GuV/G/GHG_bf.pdf

[16] Gemeinsamer Bundesausschuss. Tragende Gründe zum Beschluss des Gemeinsamen Bundesausschusses über eine Änderung der Arzneimittel-Richtlinie: Anlage III (Verordnungseinschränkungen und -ausschlüsse) – Nummer 35 (Lipidsenker) Vom 19. Dezember 2024. https://www.g-ba.de/downloads/40-268-11066/2024-12-19_AM-RL-III_Nr35-Lipidsenker_TrG.pdf

[17] National Institute for Health and Care Excellence (NICE). Cardiovascular disease: risk assessment and reduction, including lipid modification [online]. Last updated: 24 May 2023. London (GBR): NICE; 2014. (NICE Guideline; Band 181). URL: https://www.nice.org.uk/guidance/ng238

[18] Vdek. Daten zum Gesundheitswesen: Versicherte. Stand: 02.04.2024. https://www.vdek.com/presse/daten/b_versicherte.html

[19] Obermann K, Graf v. d. Schulenburg JM, Mautner GC. Ökonomische Analyse der Sekundärprävention der koronaren Herzkrankheit mit Simvastatin (Zocor) in Deutschland [Economic analysis of secondary prevention of coronary heart disease with simvastatin (Zocor) in Germany]. Med Klin (Munich). 1997 Nov 15;92(11):686–94. 9480401 10.1007/BF03044827

[20] Lauterbach KW, Gerber A, Klever-Deichert G, Stollenwerk B. Kosteneffektivität der Prävention der koronaren Herzkrankheit in Deutschland [Cost-effectiveness of prevention of coronary disease in Germany]. Z Kardiol. 2005;94 Suppl 3:III/100–4. 10.1007/s00392-005-1314-y 16258785

[21] Cook RJ, Sackett DL. The number needed to treat: a clinically useful measure of treatment effect. BMJ. 1995;310(6977):452–4. doi: 10.1136/bmj.310.6977.452 7873954 PMC2548824

[22] Byrne P, Demasi M, Jones M, Smith SM, O’Brien KK, DuBroff R. Evaluating the association between low-density lipoprotein cholesterol reduction and relative and absolute effects of statin treatment: a systematic review and meta-analysis. JAMA Intern Med. 2022;182(5):474–81. doi: 10.1001/jamainternmed.2022.0134 35285850 PMC8922205

[23] Baigent C, Keech A, Kearney P, Blackwell L, Buck G, Pollicino C, et al. Efficacy and safety of cholesterol-lowering treatment: prospective meta-analysis of data from 90,056 participants in 14 randomised trials of statins. Lancet. 2005;366(9493):1267–78. 16214597 10.1016/S0140-6736(05)67394-1

[24] Navarese EP, Robinson JG, Kowalewski M, Kolodziejczak M, Andreotti F, Bliden K, et al. Association between baseline LDL-C level and total and cardiovascular mortality after LDL-C lowering: a systematic review and meta-analysis. JAMA. 2018;319(15):1566–79. doi: 10.1001/jama.2018.2525 29677301 PMC5933331

[25] Dugré N, Lindblad AJ, Perry D, Allan GM, Braschi É, Falk J, et al. Lipid-lowering therapies for cardiovascular disease prevention and management in primary care: PEER umbrella systematic review of systematic reviews. Can Fam Physician. 2023;69(10):701–11. doi: 10.46747/cfp.6910701 37833094 PMC10575662

[26] Diederichs C, Neuhauser H, Rücker V, Busch MA, Keil U, Fitzgerald AP, et al. Predicted 10-year risk of cardiovascular mortality in the 40 to 69 year old general population without cardiovascular diseases in Germany. PLoS One. 2018;13(1):e0190441. doi: 10.1371/journal.pone.0190441 29293619 PMC5749805

[27] Kassenärztliche Bundesvereinigung. Online-Version des EBM. 1. April 2024. https://www.kbv.de/html/online-ebm.php

[28] Mach F, Baigent C, Catapano A, Koskinas K, Casula M, Badimon L, et al. 2019 ESC/EAS guidelines for the management of dyslipidaemias: lipid modification to reduce cardiovascular risk. Eur Heart J. 2020;41(1):111–88. 31504418 10.1093/eurheartj/ehz455

[29] Kolominsky-Rabas PL, Heuschmann PU, Marschall D, Emmert M, Baltzer N, Neundörfer B, et al. Lifetime cost of ischemic stroke in Germany: results and national projections from a population-based stroke registry: the Erlangen Stroke Project. Stroke. 2006;37(5):1179–83. doi: 10.1161/01.STR.0000217450.21310.90 16574918

[30] Rücker V, Heuschmann PU, O’Flaherty M, Weingärtner M, Hess M, Sedlak C, et al. Twenty-Year Time Trends in Long-Term Case-Fatality and Recurrence Rates After Ischemic Stroke Stratified by Etiology. Stroke. 2020;51(9):2778–85. doi: 10.1161/STROKEAHA.120.029972 32811383

[31] IGES. Häufigkeit und Kosten von ischämischen Schlaganfällen und Vorhofflimmern in Deutschland (GKV) unter Berücksichtigung von Versorgungsaspekten. Berlin: IGES; 2023.

[32] Brüggenjürgen B, Rupprecht H-J, Willich SN, Spannagl M, Ehlken B, Smala A, et al. Cost of atherothrombotic diseases—myocardial infarction, ischaemic stroke and peripheral arterial occlusive disease—in Germany. J Public Health. 2005;13(4):216–24. doi: 10.1007/s10389-005-0112-3

[33] Gandjour A. A parsimonious model to validate cost-effectiveness analyses on preventive health care. BMC Health Services Research. 2021;21(1):1213. 34753466 10.1186/s12913-021-07217-2 PMC8579517

[34] Sidelnikov E, Dornstauder E, Jacob C, Maas C, Pinto L, Leidl R, et al. Healthcare resource utilization and costs of cardiovascular events in patients with atherosclerotic cardiovascular disease in Germany - results of a claims database study. J Med Econ. 2022;25(1):1199–206. doi: 10.1080/13696998.2022.2141964 36330899

[35] Grootjans-van Kampen I, Engelfriet PM, van Baal PHM. Disease prevention: saving lives or reducing health care costs?. PLoS One. 2014;9(8):e104469. doi: 10.1371/journal.pone.0104469 25116681 PMC4130534

[36] Wendland G, Klever-Deichert G, Lauterbach K. Kosteneffektivität der lipidsenkenden Therapie [Cost effectiveness of lipid lowering therapy]. Herz. 2001;26(8):552–60. doi: 10.1007/pl00002059 11820158

[37] Bundesministerium für Gesundheit. Entwurf eines Gesetzes zur Stärkung der Herzgesundheit (Gesundes-Herz-Gesetz - GHG). 14.06.2024. https://www.bundesgesundheitsministerium.de/fileadmin/Dateien/3_Downloads/Gesetze_und_Verordnungen/GuV/G/GHG_RefE_bf.pdf

[38] Gemeinsamer Bundesausschuss. Tragende Gründe zum Beschluss des Gemeinsamen Bundesausschusses über eine Änderung der Arzneimittel-Richtlinie: Anlage XII - Nutzenbewertung von Arzneimitteln mit neuen Wirkstoffen nach § 35a des Fünften Buches Sozialgesetzbuch (SGB V) und Anlage XIIa? Kombinationen von Arzneimitteln mit neuen Wirkstoffen nach § 35a SGB V. Alirocumab (Neues Anwendungsgebiet: Hypercholesterinämie,? 8 Jahre bis 17 Jahre). Vom 6. Juni 2024. https://www.g-ba.de/downloads/40-268-10538/2024-06-06_AM-RL-XII-XIIa_Alirocumab_D-1000_TrG.pdf

[39] Bundesministerium für Wohnen, Stadtentwicklung und Bauwesen. Deutschland altert in den Regionen unterschiedlich schnell. 31.12.2021. https://www.deutschlandatlas.bund.de/DE/Karten/Wer-wir-sind/030-Altersgruppen-der-Bevoelkerung.html#_lo8lwb08o

[40] Visseren F, Mach F, Smulders Y, Carballo D, Koskinas K, Bäck M, et al. 2021 ESC guidelines on cardiovascular disease prevention in clinical practice. Eur Heart J. 2021;42(34):3227–337. 34458905 10.1093/eurheartj/ehab484

[41] Gandjour A. Underuse of innovative medicines in Germany: A justification for government intervention?. Health Policy. 2018;122(12):1283–6. doi: 10.1016/j.healthpol.2018.08.009 30389185

[42] Sennfält S, Norrving B, Petersson J, Ullberg T. Long-Term Survival and Function After Stroke: A Longitudinal Observational Study From the Swedish Stroke Register. Stroke. 2019;50(1):53–61. doi: 10.1161/STROKEAHA.118.022913 30580719

[43] Lazar LD, Pletcher MJ, Coxson PG, Bibbins-Domingo K, Goldman L. Cost-effectiveness of statin therapy for primary prevention in a low-cost statin era. Circulation. 2011;124(2):146–53. doi: 10.1161/CIRCULATIONAHA.110.986349 21709063

[44] Nickolaus B, Zylka-Menhorn V. Statine als Generika: “Marktjustierung“ nach Patentablauf. Dtsch Arztebl. 2003;100(12):A-750, B-638, C-598.

[45] Michaeli DT, Michaeli JC, Boch T, Michaeli T. Cost-Effectiveness of Lipid-Lowering Therapies for Cardiovascular Prevention in Germany. Cardiovasc Drugs Ther. 2023;37(4):683–94. doi: 10.1007/s10557-021-07310-y 35015186 PMC10397126

